# Active demethylation in mouse zygotes involves cytosine deamination and base excision repair

**DOI:** 10.1186/1756-8935-6-39

**Published:** 2013-11-14

**Authors:** Fátima Santos, Julian Peat, Heather Burgess, Cristina Rada, Wolf Reik, Wendy Dean

**Affiliations:** 1 Epigenetics Programme, The Babraham Institute, Cambridge CB22 3AT, UK; 2 Laboratory of Molecular Biology, Francis Crick Avenue, Cambridge CB2 0QH, UK; 3 Centre for Trophoblast Research, University of Cambridge, Cambridge CB2 3EG, UK

**Keywords:** Epigenetic reprogramming, DNA methylation, Hydroxymethylation, AID, BER, UNG2, TET3

## Abstract

**Background:**

DNA methylation in mammals is an epigenetic mark necessary for normal embryogenesis. During development active loss of methylation occurs in the male pronucleus during the first cell cycle after fertilisation. This is accompanied by major chromatin remodelling and generates a marked asymmetry between the paternal and maternal genomes. The mechanism(s) by which this is achieved implicate, among others, base excision repair (BER) components and more recently a major role for TET3 hydroxylase. To investigate these methylation dynamics further we have analysed DNA methylation and hydroxymethylation in fertilised mouse oocytes by indirect immunofluorescence (IF) and evaluated the relative contribution of different candidate factors for active demethylation in knock-out zygotes by three-dimensional imaging and IF semi-quantification.

**Results:**

We find two distinct phases of loss of paternal methylation in the zygote, one prior to and another coincident with, but not dependent on, DNA replication. TET3-mediated hydroxymethylation is limited to the replication associated second phase of demethylation. Analysis of cytosine deaminase (AID) null fertilised oocytes revealed a role for this enzyme in the second phase of loss of paternal methylation, which is independent from hydroxymethylation. Investigation into the possible repair pathways involved supports a role for AID-mediated cytosine deamination with subsequent U-G mismatch long-patch BER by UNG2 while no evidence could be found for an involvement of TDG.

**Conclusions:**

There are two observable phases of DNA demethylation in the mouse zygote, before and coincident with DNA replication. TET3 is only involved in the second phase of loss of methylation. Cytosine deamination and long-patch BER mediated by UNG2 appear to independently contribute to this second phase of active demethylation. Further work will be necessary to elucidate the mechanism(s) involved in the first phase of active demethylation that will potentially involve activities required for early sperm chromatin remodelling.

## Background

DNA methylation is an important epigenetic mark involved in gene silencing, X chromosome and transposon inactivation, genomic imprinting, and chromosome stability. DNA methylation is subject to reprogramming during development, involving both demethylation (active and passive) and *de novo* methylation phases. To date, the most clear examples of active DNA demethylation take place during the very early steps of mammalian development, namely in the zygote where the paternal genome undergoes a massive wave of loss of 5-methylcytosine (5mC) right after fertilisation [1-4].

Within 1 h of fertilisation, the paternal genome goes through major chromatin remodelling, loses protamines and is re-packaged by maternal nucleosomal histones, forming the paternal pronucleus [5,6]. Post-fertilisation development can be defined by the pronuclear stages PN0/1 to PN5; PN0-PN2 embryos are in the G1 phase, PN3 and PN4 embryos are largely in S phase, replicating both the paternal and maternal genomes, and PN5 embryos are mostly in the post-replicative G2 phase [3,7-9]. Several reports have shown that the paternal genome undergoes genome-wide DNA demethylation via an active mechanism before replicating its DNA [1-4]. The search for enzymes responsible for this demethylation has produced numerous candidates and reaction mechanisms [10-13]. These fall within three main groups: (1) direct removal of the methyl group from the 5-C position of cytosine; (2) DNA repair, either base excision repair (BER) or nucleotide excision repair (NER); and (3) iterative enzymatic oxidation leading to conversion of 5mC to 5- hydroxymethylcytosine (5hmC), 5-formylcytosine (5fC), and 5-carboxylcytosine (5caC).

The methyl-CpG-binding domain protein 2 (MBD2) was reported to possess direct demethylase activity [14], but the result could not be reproduced by others. DNA glycosylases have been described in plants that can remove 5mC, leaving an abasic site that is repaired by the BER machinery but mammalian glycosylases (e.g., thymine DNA glycosylase (TDG) and methyl-CpG-binding domain protein 4 (MBD4)) show weak activity on 5mC *in vitro*[15]. However, both MBD2 and MBD4 null fertilised oocytes undergo paternal loss of DNA methylation indistinguishable from matched controls [16].

An alternative to direct removal of 5mC by a DNA glycosylase is enzymatic deamination of 5mC to thymine, followed by T-G mismatch specific BER that replaces thymine with cytosine [17]. Two classes of enzymes have been proposed to be capable of carrying out the first step in this process: cytosine deaminases and DNA methyltransferases (reviewed in [11]). Cytosine DNA deaminases convert cytosine to uracil in nucleic acids and are well known from their roles in RNA editing, viral defence and antibody diversification [18]. Recently a series of results have pointed to an involvement of activation-induced deaminase (AID) mediated cytosine deamination in DNA demethylation in primordial germ cells (PGCs) and induced pluripotent stem (iPS) cell reprogramming, in cancer and embryonic stem (ES) cell gene expression [19-21]. Furthermore, overexpression of AID and MBD4 have been described to cause general demethylation of the zebrafish embryo genome, suggesting that deamination of 5mC followed by BER of T-G mismatches results in demethylation [22]. Gadd45, a p53-inducible gene involved in a variety of cellular processes, seems to facilitate this process and it has also been shown to interact with nucleotide excision repair (NER) components [22,23]. Models have been proposed for Gadd45 mediated demethylation of DNA either by deamination followed by BER or NER, or even by a combination involving consecutive NER and BER mechanisms (reviewed in [24]). AID's best defined activity is in B lymphocytes, where deamination of cytosines leading to uracil initiates both somatic hypermutation and immunoglobin class switch recombination [25-27]. However, its expression in mouse oocytes as well as in ES cells and PGCs [28], make it a potential candidate for performing global demethylation. *In vitro* assays have shown AID has 5mC deaminase activity, resulting in thymine and, therefore, T-G mismatches in DNA, which can be effectively repaired through the BER pathway [28]. Cytosine and 5-methylcytosine can also be enzymatically deaminated by DNA methyltransferases (DNMTs), primarily known as enzymes that transfer a methyl group to the C-5 position of cytosine from the methyl donor S-adenosylmethionine (SAM- reviewed in [29]). Recent work in mammalian cell lines has led to the proposal that deamination by the Dnmt3a and Dnmt3b DNA methyltransferases could be a means of achieving fast, active DNA demethylation at promoters undergoing transcriptional cycling, by generating thymine, which is repaired via TDG and other enzymes [12,30-32]. More recently, studies have suggested that loss of DNA methylation in the paternal genome in the zygote is primarily dependent on TET3 [4,33], a member of the ten-eleven translocation (TET) family of DNA dioxygenases, which are capable of converting 5mC to 5- hydroxymethylcytosine (5hmC), 5-formylcytosine (5fC) and 5-carboxylcytosine (5caC) through iterative oxidation [34], suggesting that the main mechanism involved in active genome-wide demethylation is via oxidation of 5mC. Although these various models for active loss of DNA methylation from the paternal pronucleus have been proposed, they have, for the most part, overlooked the evidence that this process occurs in two phases - before and coincident with DNA replication. To investigate this DNA methylation dynamics we have analysed fertilised mouse oocytes, by indirect immunofluorescence (IF) of DNA methylation and hydroxymethylation, and evaluated the relative contribution of different activities to active demethylation, by three-dimensional (3D) imaging and IF semi-quantification.

## Results and discussion

We have previously characterised the dynamics of DNA methylation loss during the first cell cycle in the mouse by indirect immunofluorescence [3,35]. Since then, several similar studies have been published, more recently including other modifications of DNA methylation, namely hydroxymethylation, and attempts have been made to semi-quantify the immunofluorescence signals in order to get a sense of the kinetics of demethylation [4,8,33,36,37]. These approaches have provided clear evidence for the oxidation of 5mC in the zygote, specifically in the paternal pronucleus, seemingly explaining the concomitant decrease in DNA methylation.

**Figure 1:**
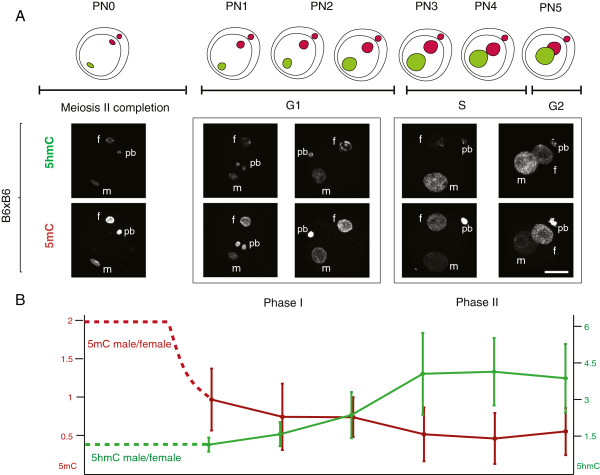
**Paternal loss of DNA methylation occurs in two phases. (A)** Diagrammatic illustration and representative two-dimensional (2D) projections of Z-stack images of control (B6 x B6) pronuclear stage embryos (PN0 to PN5) simultaneously stained for DNA methylation (5mC) and hydroxymethylation (5hmC) clearly showing two phases of paternal loss of methylation, Phase I, corresponding to a pre-replicative state with no observable change in DNA hydroxymethylation, and Phase II, when DNA replication is taking place, and during which a very significant increase in DNA hydroxymethylation in the paternal pronucleus takes place. Scale bar 25 μm. f, female pronucleus; m, male pronucleus; pb, polar body. **(B)** The dynamic changes of DNA methylation and hydroxymethylation during the first cell cycle can be represented by the ratio of the total immunofluorescence signal (3D imaging) between the maternal and paternal pronuclei (male/female ratio) for 5mC and 5hmC, respectively. Values at time of fertilisation are hypothetical (dashed lines), calculated considering a minimum initial total 5mC and 5hmC for sperm and oocytes (see text for explanation). Values plotted for stages between PN1 and PN5 (minimum 10 embryos per stage). Bars indicate standard deviation.

**Figure 2:**
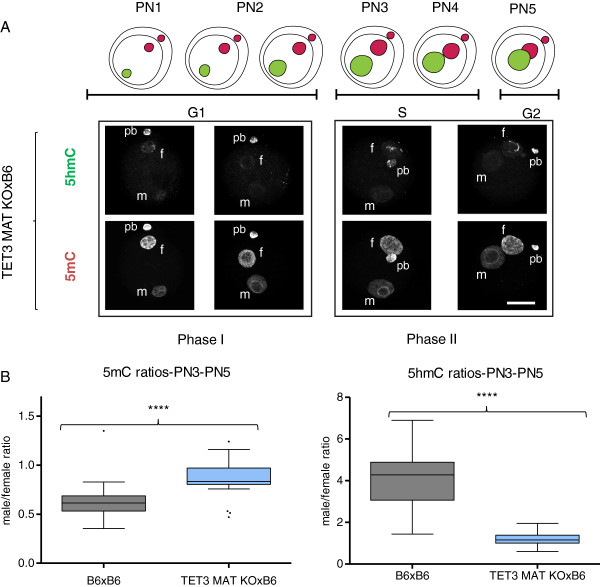
**TET3 null fertilised oocytes show increased paternal DNA methylation and reduced hydroxymethylation. (A)** Diagrammatic illustration and representative 2D projections of Z-stack images of TET3 maternally deleted oocytes fertilised by control sperm (TET3 MAT KOxB6) pronuclear stage embryos (PN1 to PN5) simultaneously stained for DNA methylation (5mC) and hydroxymethylation (5hmC) clearly showing paternal loss of methylation during Phase I, corresponding to a pre-replicative state, but no apparent further demethylation during Phase II, when DNA replication is taking place, and during which there is complete failure in TET3 null oocytes to generate DNA hydroxymethylation in the paternal pronucleus. Scale bar 25 μm. f, female pronucleus; m, male pronucleus; pb, polar body. **(B)** Comparison of the changes of DNA methylation and hydroxymethylation between control (B6xB6; Figure 1A) and TET3 maternally deleted (TET3 MAT KOxB6) mid-late zygotes. Box-and-whisker plots of the total immunofluorescence signal (3D imaging semi-quantification) ratio between the paternal and maternal pronuclei (male/female ratio) for 5mC and 5hmC, respectively, showing, on the left, a very significant increase in the levels of paternal DNA methylation and, on the right, an equally significant decrease in the levels of hydroxymethylation in TET3 maternally deleted zygotes compared to controls. ****(*P* <0.0001, two-tailed Mann–Whitney test).

**Figure 3:**
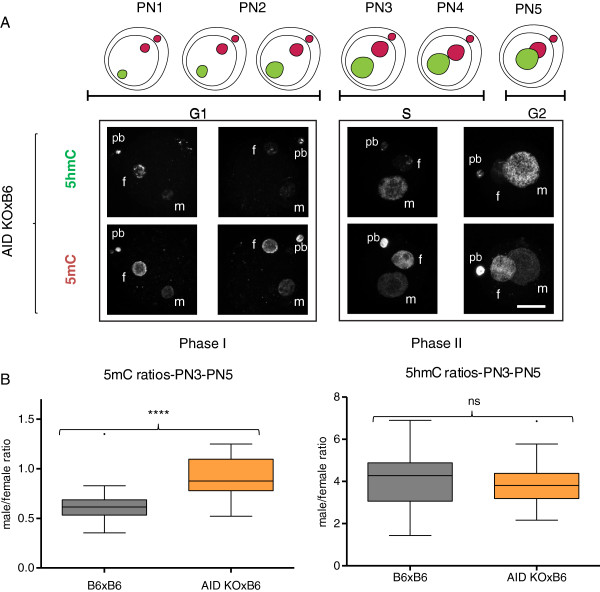
**AID null fertilised oocytes show increased paternal DNA methylation but equal levels of hydroxymethylation. (A)** Diagrammatic illustration and representative 2D projections of Z-stack images of AID null oocytes fertilised by control sperm (AID KOxB6) pronuclear stage embryos (PN1 to PN5) simultaneously stained for DNA methylation (5mC) and hydroxymethylation (5hmC) clearly showing paternal loss of methylation during Phase I, corresponding to a pre-replicative state, but no apparent further demethylation during Phase II, when DNA replication is taking place, while showing a striking increase in DNA hydroxymethylation in the paternal pronucleus, similar to that observed in control (B6xB6) embryos. Scale bar 25 μm. f, female pronucleus; m, male pronucleus; pb, polar body. **(B)** Comparison of the changes of DNA methylation and hydroxymethylation between control (B6xB6) and AID null (AID KOxB6) mid-late zygotes. Box-and-whisker plots of the total immunofluorescence signal (3D imaging semi-quantification) ratio between the paternal and maternal pronuclei (male/female ratio) for 5mC and 5hmC, respectively, showing, on the left, a very significant increase in the levels of paternal DNA methylation but no significant difference in the levels of hydroxymethylation in AID null zygotes compared to controls, on the right. ****(*P* <0.0001, two-tailed Mann–Whitney test), ns (*P* = 0.6330, two-tailed unpaired t test with Welch’s correction).

**Figure 4:**
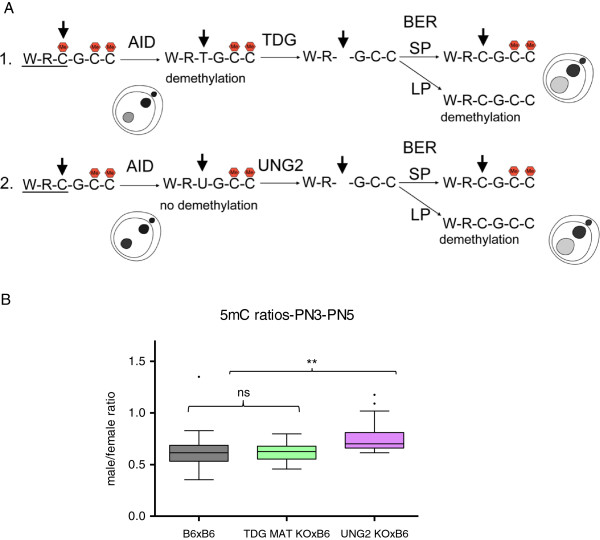
**Evidence supports AID mediated cytosine deamination with U-G mismatch long-patch BER in the zygote. (A)** Schematic representation of the possible AID-mediated deamination scenarios. (1) AID deamination of a methylated cytosine in the context of the preferred binding motif (WRC) resulting in demethylation and creating a T-G mismatch. This would be recognised by TDG generating and apyrimidinic site that would subsequently be repaired by either short-patch (SP) (no further loss of methylation in neighbouring methylated cytosines) or long-patch (LP) (possible further demethylation by new incorporation of cytosines not followed by *de novo* methylation) BER. (2) AID deamination of a non-methylated cytosine in the context of the preferred binding motif (WRC) resulting in no loss of methylation and creating a U-G mismatch. This would be recognised by UNG2 generating and apurinic site that would subsequently be repaired by either SP (no loss of methylation in neighbouring methylated cytosines) or LP (resulting in demethylation by new incorporation of cytosines not followed by *de novo* methylation) BER. **(B)** Comparison of the changes of DNA methylation between wild-type control (B6xB6), TDG maternally deleted (TDG MAT KOxB6) and UNG2 null (UNG2 KOxB6) late zygotes. Box-and-whisker plots of the total immunofluorescence signal (3D imaging semi-quantification) ratio between the paternal and maternal pronuclei (male/female ratio) for 5mC showing there is only a significant increase in the levels of paternal DNA methylation in UNG2 deleted and not in TDG maternally deleted fertilised oocytes. This is compatible with AID-mediated cytosine deamination followed by LP BER with no evidence for direct 5mC deamination and T-G mismatch repair, which is in agreement with the reported reduced activity of AID on 5mC relative to cytosine, its canonical substrate. **(*P* < 0.05, ANOVA, Dunn’s multiple comparison test), ns (*P* > 0.05, ANOVA, Dunn’s multiple comparison test).

We have established a protocol that allows us to reproducibly semi-quantify the staining levels of both DNA methylation and hydroxymethylation in pre-implantation embryos (see Methods and Additional files 1 and 2) and have used it to validate qualitative immunofluorescence results suggesting that, by the time the fertilised oocyte enters G1 a substantial amount of DNA methylation signal has already been lost from the paternal pronucleus, prior to the increase in the observed levels of hydroxymethylation (Figure 1A). To probe the kinetics of these two processes (loss of 5mC and increase in 5hmC) we have captured 3D data of fertilised oocytes simultaneously stained for 5mC and 5hmC. The signal intensity of both modifications was semi-quantified and the ratio between the paternal and maternal pronuclei in each individual one-cell embryo (from PN1 to PN5) calculated. The initial values for the two complements cannot be measured using this protocol as condensed sperm is notably impervious to immunostaining due to its highly compacted chromatin state [6], resulting in unreliable semi-quantification values for PN0. Based on the levels of DNA methylation for mouse sperm and oocytes, reported by genome-wide or reduced representation bisulphite-sequencing, and despite variability in the quantification methods, there is a general agreement that DNA methylation levels in sperm are twice that observed in the oocyte [38,39]. It is therefore possible to calculate a conservative (using the lowest estimate based on whole-genome bisulphite-sequencing, [38]) theoretical paternal to maternal ratio of DNA methylation of 2 for PN0 (Figure 1B). Functional grouping of the data according to cell cycle [8], corresponding to embryos in G1 (Early; PN1-PN2) and in S-G2 phase (Mid-Late; PN3-PN5), was used to compare our data with that collected by others. A summary of paternal to maternal ratios of 5mC levels based on reports from the literature, as well as this work, is presented in Table 1. From its examination it becomes clear that, in all cases and consistently throughout the data generated by multiple independent labs, the paternal/maternal ratio of DNA methylation in G1 is never close to the theoretical initial PN0 value of 2, predicted from the 5mC levels reported for the gametes. There is thus an observable first loss of paternal DNA methylation levels of approximately 60% by the time the zygote enters G1 (average ratio drop from 2 to 0.8) and a further loss of 20% (average ratio drop from 0.8 to 0.5) thereafter. This supports the distinction between two phases of active demethylation, one in G1 and the other in S-G2 (Figure 1A, B). In contrast, the levels of hydroxymethylation, evaluated the same way, show a rather different profile. While the paternal/maternal ratio remains relatively stable during Phase I, there is a remarkable increase during Phase II with a marked rise in the 5hmC signal in the paternal pronucleus (Figure 1A, B). Although concomitant with S-phase, this increase in paternal hydroxymethylation is not dependent on DNA replication ([4] and Additional file 3). This is again in agreement with our analysis of results reported in the literature, which shows a significant increase in the 5hmC paternal/maternal ratio occurring between Early (Phase I) and Mid-Late (Phase II) one-cell embryos (Table 2). The changes in 5hmC signal point to the involvement of TET-mediated 5mC oxidation during Phase II, but not Phase I, of paternal loss of DNA methylation. In order to test this hypothesis, we used a conditional (Zp3-Cre) TET3 knock-out mouse generated in the lab. TET3 null oocytes were fertilised by C57Bl/6 J (B6) sperm and the same simultaneous 5mC and 5hmC staining and semi-quantification analysis was performed (Figure 2). The results confirmed the dependency of paternal hydroxymethylation on the maternally derived TET3 protein [33] with no observable increase in 5hmC staining in Mid-Late one-cell embryos (Figure 2A) and consequently a very significant difference in the paternal/maternal ratios between TET3 MAT KO and control B6 fertilised oocytes (Figure 2B). The absence of paternal 5hmC signal increase was accompanied by an equally significant rise in paternal DNA methylation staining levels in PN3-PN5 one-cell embryos (Figure 2B), supporting a role for TET3-mediated hydroxymethylation in the loss of paternal DNA methylation at this stage. We then concentrated our attention on the first phase of paternal DNA methylation loss that seems to be independent of hydroxymethylation. DNA repair has been proposed as a mechanism to explain active DNA demethylation and there is evidence for DNA repair pathways being involved in paternal DNA methylation loss in the zygote, particularly BER [11,40,41]. Moreover, deamination has been implicated as a possible upstream event initiating the BER mediated demethylation [42]. Activation-induced cytidine deaminase has also been shown to be capable of deaminating 5mC and is expressed in pluripotent tissues, including mouse oocytes [28]. AID was initially thought to be only relevant in B-lymphocytes, where it is essential for somatic hypermutation and class-switch recombination [26,27]. Recently AID has been shown to be involved in dynamic methylation changes in a variety of tissues ranging from PGCs [21] to ES and iPS cells [20,43]. The presence of AID protein in control mouse oocytes was confirmed by indirect immunofluorescence (Additional file 4). Making use of the same AID knock-out mouse model that showed altered DNA methylation levels in PGCs [21], AID null oocytes were fertilised by either AID (Additional file 5) or B6 (Figure 3) sperm and the same simultaneous 5mC and 5hmC staining and semi-quantification analysis was performed. A significant difference was found in the paternal/maternal ratio of DNA methylation between AID and control (B6) fertilised oocytes, with no apparent effect on the levels of hydroxymethylation (Figure 3B). Notably, this difference was only detectable in PN3-PN5 (Phase II) embryos and no difference could be seen in PN1-PN2 (Phase I) zygotes (Figure 3A and Additional file 5) despite reports indicating that AID works during G1 [44]. AID deaminates cytosines at immunoglobulin genes on single-stranded DNA thought to be made available during transcription [45], however post-fertilisation mouse oocytes are transcriptionally silent [46] thus necessitating other means of generating single-stranded DNA substrates. As such, it is reasonable to expect that AID might be able to deaminate immediately after fertilisation during nucleoprotamine exchange, a time when single-stranded DNA would be accessible. Recently it has been described that, in both class-switching B-cells and *E. coli*, negative DNA supercoiling, and hence generation of single-stranded DNA, enhances AID mutagenesis [47]. Within 1 h after fertilisation the paternal chromatin suffers a complete remodelling resulting in an exchange of the sperm protamines by maternally derived histones [5,6]. This process will likely involve the generation of DNA supercoils, mirroring what happens in the course of nucleosome elimination and incorporation of protamines during spermiogenesis [48], which may facilitate AID mediated deamination. Deamination of 5mC during G1 in the fertilised oocyte would immediately result in loss of DNA methylation immunofluorescence signal, given the high specificity of the antibody used, leading to an expected increase of the paternal/maternal 5mC ratio in PN1-PN2 (G1) staged zygotes compared to B6 controls. The fact that no significant difference could be found suggests that no such deamination has occurred in the control zygotes. The results obtained were therefore surprising. Investigating the known mechanisms for DNA repair following AID-mediated deamination provided a possible explanation (Additional file 6). Repair can be achieved by different mechanisms, both error-prone and error-free [49]. Deamination of 5mC generates T creating a T-G mismatch which is the preferred substrate for TDG and MBD4 glycosylases [28,50]. On the other hand, deamination of C generates U and uracil residues in DNA are also largely resolved by BER, with the uracil being removed by a DNA glycosylase [50]. Uracyl glycosylase (UNG2) has been implicated as the major glycosylase responsible for repair of C to U mismatches following deamination [51]. BER enables the repair of damaged DNA via two general pathways, short-patch and long-patch [50,52,53]. Short-patch (SP) BER replaces a single nucleotide by polymerase β and the newly synthesized DNA is sealed by DNA ligase III/X-ray cross-complementing group 1 (XRCC1) heterodimer. Long-patch (LP) BER inserts two to 13 nucleotides by coordinate action of polymerase δ, proliferating cell nuclear antigen (PCNA), flap endonuclease 1 and DNA ligase I. Although at face value only the deamination of 5mC could result in demethylation, in the case of cytosine deamination followed by LP BER, there would be the possibility for any methylated cytosines adjacent to the U-G mismatch to be replaced by newly incorporated non-modified cytosines and the original methylated state would be lost, resulting in demethylation (Additional file 6). It is thus possible to achieve DNA methylation loss through a C to U deamination and subsequent replacement of adjacent methylated cytosines by LP BER (Figure 4A). To resolve these potential alternative downstream pathways, analysis of knock-out mouse oocytes for either TDG [54] or UNG2 [55], fertilised by B6 sperm, was performed (Figure 4B). The results support a role for AID mediated cytosine deamination with subsequent U-G mismatch LP BER and no evidence could be found for direct 5mC deamination and T-G mismatch repair. This is in agreement with the reported reduced activity of AID on 5mC when compared to cytosine, its canonical substrate [10,28]. Furthermore, PARP1, a hallmark of LP BER [52,53] (Additional file 6), has been found to be predominantly confined to the paternal pronucleus in PN3-PN5 staged zygotes [40,41] and its specific inhibition caused a significant increase in the paternal/maternal ratio of DNA methylation compared to controls [40]. In this scenario, AID-mediated cytosine deamination can still occur in G1 (PN1-PN2), without causing any immediate 5mC loss, and the resulting U-G mismatches could later be repaired by LP BER, presumably causing loss of adjacent methylated cytosines with consequent demethylation in PN3-PN5 staged zygotes. Although speculative, this model can fully account for the results obtained.

**Table 1:** DNA methylation (5mC) average paternal to maternal ratios (indirect immunofluorescence)

**Study**	**Early (PN1-PN2)**	**Mid-Late (PN3-PN5)**	**Mouse strain**
Iqbal et al., 2011^1^	0.9	0.47	FVB
Wossidlo et al., 2011^1^	0.53	0.34	BDF1 (C57BL/6 x DBA)
Inoue and Zhang, 2011	0.85	0.61	BDF1 (C57BL/6 x DBA)
Salvaing et al., 2012^1^	0.72	0.47	F1 (C57BL/6 x CBA)
This study	0.84	0.62	C57BL/6 J

^1^DNA staining was used for normalisation of paternal/maternal ratios.

**Table 2:** DNA hydroxymethylation (5hmC) average paternal to maternal ratios (indirect immunofluorescence)

**Study**	**Early (PN1-PN2)**	**Mid-Late (PN3-PN5)**	**Mouse strain**
Iqbal et al., 2011^1^	0.78	2.82	FVB
Wossidlo et al., 2011^1^	0.85	2.44	BDF1 (C57BL/6 x DBA)
Inoue and Zhang, 2011	1.89	4.98	BDF1 (C57BL/6 x DBA)
Salvaing et al., 2012^1^	0.9	2.23	F1 (C57BL/6 x CBA)
This study	1.334	4.01	C57BL/6 J

^1^DNA staining was used for normalisation of paternal/maternal ratios.

According to recent reports, deamination of cytosine and 5-methylcytosine could also be mediated by DNA methyltransferases, namely the Dnmt3a and Dnmt3b *de novo* methyltransferases (reviewed in [11,12]) and more recently that DNA could be directly demethylated by the same enzymes, at least in an *in vitro* system [56]. Moreover, results from ES cells suggest that TET and Dnmt3 enzymes may interact at common binding sites [57]. These prompted us to investigate whether Dnmt3a null oocytes were capable of demethylating a control (B6) sperm using a conditional (Zp3-Cre) Dnmt3a knock-out mouse model [58]. Oocytes null for Dnmt3a are severely depleted of DNA methylation [59] which precluded the use of the paternal/maternal ratio as a measure of demethylation as the maternal pronucleus showed only residual levels of 5mC staining (Additional file 7A); instead we directly compared the total levels of immunofluorescence signal in the paternal pronuclei of Dnmt3a null, control (B6) and AID null oocytes fertilised by B6 sperm (Additional file 7B). No significant difference, in either 5mC or 5hmC levels, between paternal pronuclei in control and Dnmt3a null fertilised oocytes was observed, whereas both are significantly different from AID null in 5mC but not in 5hmC levels (Additional file 7B). These results do not support a role for Dnmt3a in paternal active demethylation in the mouse one-cell embryo.

As a whole, our results support a role for both 5mC hydroxymethylation and cytosine deamination, followed by LP BER, as demethylation mechanisms in the mouse zygote. Both these mechanisms seem to contribute independently to a decrease in paternal DNA methylation coincident, but not reliant, on DNA replication. Furthermore, an initial loss of paternal DNA methylation, prior to S-phase, seems to take place, for which none of the activities investigated in this work seem to supply an explanation. It is likely that this early demethylation is related to the need for major chromatin remodelling of the sperm within the first hours post fertilisation and may involve other pathways of DNA repair. NER has been suggested as a candidate for DNA demethylation (reviewed in [10,11]), removes bulky DNA lesions and is a multistep process involving the action of as many as 20 to 30 proteins working in a well-defined sequence [60]. Other repair pathways, such as non-homologous end joining (NHEJ) and homologous recombination (HR), could also play a role in this first phase of paternal loss of methylation [61]. The complexity, and possible redundancy, of these alternative pathways will require considerable research effort in order to elucidate this early DNA methylation loss.

## Conclusions

Our analysis shows that there are two observable phases of active DNA demethylation in the mouse zygote, before and coincident with DNA replication. We further show that TET3 seems to be involved only in the second phase of loss of methylation. Cytosine deamination and uracil BER seem to also independently contribute to this second phase of active demethylation. Together these findings allow us to conclude that demethylation is achieved by at least two parallel mechanisms which may or may not be partially redundant. Our results highlight the dynamic nature of DNA demethylation, with two apparent distinct stages (Phase I and Phase II). The major increase in 5hmC (Phase II) seems to be uncoupled from the initial loss of DNA methylation (Phase I) which was not previously acknowledged. Still, further work will be necessary to elucidate the mechanism(s) involved in the first phase of demethylation, likely to involve activities required for early chromatin remodelling on fertilisation and perhaps other types of DNA repair.

To our knowledge, this is the first report of possible involvement of LP BER in DNA demethylation, opening new avenues of investigation not formerly considered.

## Methods

### Mice and sample collection

All experimental procedures were approved by the Animal Welfare, Experimentation and Ethics Committee (AWEEC) at the Babraham Institute and were performed under licenses by the Home Office (UK) in accordance with the Animals (Scientific Procedures) Act 1986.

Fertilised mouse oocytes were collected on the day of plugging from naturally mated inbred C57BL/6 J (B6) mice supplied from breeding colonies in the Biological Support Unit at the Babraham Institute. Mechanistic investigation into the loss of DNA methylation was made possible through the generation of conditional deletion of key activities, Dnmt3a [62] and TDG [54] (rescued by a floxed TDG minigene), derived by breeding female mice homozygous for floxed alleles together with the Zp3 Cre recombinase transgene [63]. Conditionally deleted oocytes generated in this way were referred to as maternal knock-outs (MAT KO). In all cases these MAT KO generating females were bred to wild-type B6 control males. Constitutively deleted activities were derived for AID [26] and UNG2 [51,55] from homozygous females null for the respective enzymes.

Individual zygote pronuclear staging was performed as previously described [3]. The cell-cycle state of these different stages has been characterised according to the literature, establishing that zygotes between PN1 and PN2 will be in G1 and from PN3 to PN5 in S-G2 [8].

### Generation of TET3 conditional deletion

C57BL/6 N Tac ES cells (TaconicArtemis) were targeted with a vector introducing LoxP sites around exon 5 of Tet3 RefSeq NM_183138.2 (sequence: CCGGACCTGTGCTTGCCAAGGCAAAGACCCTAACACCTGCGGTGCCTCCTTCTCCTTCGGCTGTTCCTGGAGCATGTACTTCAACGGCTGCAAATATGCTCGGAGCAAGACGCCACGAAAGTTCCGCCTCACGGGAGACAATCCGAAGGAG) which encodes residues required for chelation of Fe(II) and is upstream of exons containing other key catalytic residues [64]. Expression of Cre recombinase results in excision of this region and a frame-shift from exon 6 affecting all downstream exons until a premature stop codon in exon 7. Animals heterozygous for the floxed allele were bred to a transgenic mouse line containing Zp3 Cre on a B6 background [63] and homozygous mice were generated by inter-crossing to give females of the appropriate genotype.

### Immunofluorescence and confocal microscopy

Antibody staining of DNA methylation (Eurogentec, BI-MECY) and hydroxymethylation (Active Motif, 39769) was performed as previously described [9] with modifications. Briefly, zygotes were fixed with 4% PFA for 15 min and, after permeabilisation with 0.5% Triton X-100, the samples were treated with 4 N HCl for 10 min at room temperature, washed in PBS/Tween and blocked overnight; simultaneous incubation with both primary antibodies followed by simultaneous secondary detection (AlexaFluor, Molecular probes, Invitrogen) was used. To allow for full 3D sample capture the samples were mounted in fibrin clots [65]. Image acquisition was performed with a LSM 510 Meta confocal laser scanning microscope (Carl Zeiss) equipped with a ‘Plan-Apochromat’ 63x/1.40 DIC oil-immersion objective and an Olympus FV1000 equipped with a UPLSAPO 60x/1.35 DIC oil-immersion objective. DNA was counterstained with YOYO1™ (Molecular Probes, Life Technologies). Z-stacks of 20 to 65 optical sections were collected from each zygote (700x700, pixel size; z-step, 0.50 μm). At least 10 zygotes of each group were imaged from at least two biological replicates. Images were pseudo-coloured using Adobe Photoshop CS4.

### Semi-quantification and statistics

Fluorescence semi-quantification analysis (total sum, 3D rendering) was performed as follows, 3D reconstruction of confocal image stacks was performed using Volocity 5.5 (Improvision), after which regions of interest (ROIs) were defined around each pronucleus and total voxel count signal intensity (SUM) for each channel was computed. Examples of each of these steps can be found in Additional file 1. The data were then exported into Excel and the individual maternal to paternal ratios (male/female ratios) for each zygote calculated as this measure is widely used in the field and allows for a good degree of comparison between studies. Statistical analysis (analysis of variance (ANOVA), Mann–Whitney or unpaired t test) and whisker-plot graphs were performed with GraphPad Prism 5 and 6.

## Abbreviations

2D: Two-dimensional; 3D: Three-dimensional; 5caC: 5-carboxylcytosine; 5fC: 5-formylcytosine; 5hmC: 5-hydroxymethylcytosine; 5mC: 5-methylcytosine; AID: Activation-induced deaminase; ANOVA: Analysis of variance; APE1: Apurinic-apyrimidinic endonuclease 1; B6: C57BL/6 J mouse strain; BER: Base excision repair; C: Cytosine; DNA: Deoxyribonucleic acid; ES cells: Embryonic stem cells; Dnmt3a: DNA (cytosine-5)-methyltransferase 3a; Dnmt3b: DNA (cytosine-5)-methyltransferase 3b; G: Guanosine; HCl: Hydrochloric acid; HR: Homologous recombination; IF: Immunofluorescence; iPS cells: induced pluripotent stem cells; LP: Long-patch; MAT KO: Maternal knock-out; MBD2: Methyl-CpG binding Domain 2; MBD4: Methyl-CpG binding Domain 4; NER: Nucleotide excision repair; NHEJ: Non-homologous end joining; PARP1: poly-ADP-ribose polymerase 1; PCNA: Proliferating cell nuclear antigen; PGCs: Primordial germ cells; ROIs: Regions of interest; SP: Short-patch; T: Thymine; TDG: Thymine DNA glycosylase; TET3: Ten-eleven translocation 3; U: Uracil; UNG2: Uracil-DNA glycosylase 2; XRCC1: X-ray cross-complementing group 1.

## Competing interests

The authors declare that they have no competing interests.

## Authors’ contributions

FS, WD and WR generated the main idea of the work and developed the study design, both conceptually and methodologically. WD organised and collected the samples. FS acquired and analysed the data. FS, WD and WR contributed to analysis and interpretation of the data. CR contributed materials. JP and HB were responsible for mouse genotyping of TET3 and TDG strains, respectively. FS, WD and WR wrote the manuscript. FS, WD, JP, HB, CR and WR made comments, suggested appropriate modifications and made corrections. All authors read and approved the final manuscript.

## Authors’ information

Wolf Reik and Wendy Dean are senior authors.

## Supplementary Material

Additional file 1 **3D reconstruction of confocal image stacks and total fluorescence semi-quantification.** Volocity 5.5 (Improvision) was used for 3D rendering and signal semi-quantification of each individual embryo Z-stack. (A) Screen-shot showing an XYZ view of a representative Z-stack. (B) Screen-shot showing the 3D rendering of the same Z-stack. (C) Screen-shot showing the regions of interest (ROIs) defined around each of the objects inside the sample, paternal pronucleus (red), maternal pronucleus (green) and polar body (blue). (D) Screen-shot showing the protocol to define the ROIs and subsequent computation of several measurements, including Sum signal intensity for each of the channels, used as a measure of the total signal for 5hmC (Channel: 2) and 5mC (Channel: 3). Click here for file

Additional file 2 **Fluorescence semi-quantification protocol validation data.** (A) Four independent samples of B6xB6 generated fertilised oocytes between PN3 and PN5 were evaluated using the optimized protocol for simultaneous staining of 5mC and 5hmC, 3D image acquisition and semi-quantification. Statistical analysis (ANOVA) shows no significant differences can be found between the four replicates. (B) Mouse embryonic stem cells (E14) were cultured in both serum and 2i conditions [57] and analysed for global DNA methylation levels by using the optimised immunofluorescence semi-quantification protocol (left) or by mass-spectrometry (right). Both methods are in agreement both qualitatively (E14 serum > E14 2i) and quantitatively (E14 serum 40% to 50% more methylated than E14 2i). Click here for file

Additional file 3 **Replication inhibition does not affect DNA methylation or hydroxymethylation paternal/maternal ratios in the zygote.** Two independent replicates of at least 15 B6xB6 early fertilised oocytes were collected and incubated in M16 medium (M-7292-SIGMA) supplemented with either 2.5 μL/mL DMSO (control) or 2.5 μL/mL Aphidicolin (A4487-SIGMA) and cultured (37°C; 5%CO_2_) for 5 h. For replication analysis both groups (control-DMSO and Aphidicolin) were then transferred to a fresh same composition medium drop, to which 20 μM EdU (Click-iT™ EdU Alexa Fluor® 488, Invitrogen) was added, for a further 1 h (detection according to the manufacturer’s instructions). (A) Representative images of control (DMSO) and replication inhibited (Aphidicolin) embryos. Single optical slices. EdU-green; 5hmC-red; 5mC-white. Scale bar 25 μm. f, female pronucleus; m, male pronucleus; pb, polar body. (B) Comparison of the changes of DNA methylation and hydroxymethylation between control (DMSO) and replication inhibited (Aphidicolin) mid-late zygotes. Box-and-whisker plots of the total immunofluorescence signal (3D imaging semi-quantification) ratio between the paternal and maternal pronuclei (male/female ratio) for 5mC and 5hmC, respectively, showing no significant difference (unpaired t test) in the levels of methylation (left, *P* = 0.7842) or hydroxymethylation (right, *P* = 0.0748) in Aphidicolin-treated zygotes compared to controls (DMSO). Click here for file

Additional file 4 **AID is expressed in mouse oocytes and localises to the pronuclei.** Wild-type controls (B6xB6) and AID null (AID KO x (C57Bl/6JxCBA)-F1) zygotes were stained with an antibody against AID (A-15, Santa Cruz Biotechnology) and DAPI. The control zygotes show a typical predominantly cytoplasmic localisation of AID protein, as has been described for B-cells (reviewed in [66]), but there is visible signal in both pronuclei that is completely absent in the AID null fertilised oocytes. Scale bar 25 μm. f, female pronucleus; m, male pronucleus; pb, polar body. Click here for file

Additional file 5 **AID null zygotes show no significant 5mC difference during Phase I of paternal demethylation.** Comparison of the changes of DNA methylation and hydroxymethylation between wild-type control (B6xB6) and AID null (AIDxAID) Phase I (PN1-PN2) and Phase II (PN3-PN5) zygotes. Box-and-whisker plots of the total immunofluorescence signal (3D imaging semi-quantification) ratio between the paternal and maternal pronuclei (male/female ratio) for 5mC and 5hmC, respectively, showing, on the left, a very significant increase in the levels of paternal DNA methylation in Phase II but no significant difference in Phase I fertilised oocytes. No significant differences could be found in the levels of hydroxymethylation in AID null compared to wild-type in either Phase I or Phase II zygotes (on the right). ****(*P* <0.0001,Mann–Whitney test), ns (*P* >0.05, Unpaired t test). Click here for file

Additional file 6 **AID-mediated deamination and BER SP and LP pathways.** Diagram of the SP and LP BER pathways for AID-mediated DNA deamination. AID has been shown *in vitro* and in *E. coli* to be capable of deaminating 5-methylcytosine (5mC), generating T-G mismatches and thus directly removing methylation from DNA (left hand-side), although the preferred substrate is cytosine, very efficiently generating U-G mismatches but having no direct effect on DNA methylation loss (right hand-side). After removal of the mismatched base (black circles) by a DNA glycosylase (TDG in the case of a T-G mismatch and UNG2 in the case of an U-G mismatch) and incision by apurinic/apyrimidinic endonuclease (APE1), BER may proceed by the SP repair or by the LP repair. SP BER replaces a single nucleotide by polymerase β and the newly synthesized DNA sealed by DNA ligase III/X-ray cross-complementing group 1 (XRCC1) heterodimer. LP BER inserts two to 13 nucleotides by concordant action of polymerase δ, proliferating cell nuclear antigen (PCNA), flap endonuclease 1 and DNA ligase I. In this case, any methylated cytosines adjacent to the generated U-G mismatch would be replaced by new cytosines and, if not subsequently *de novo* methylated, the original methylated state would be lost resulting in demethylation. Poly ADP Ribose Polymerase 1 (PARP1), which binds to and is activated by DNA strand breaks, has been implicated in LP repair promoting the rapid recruitment of PAR-binding proteins to the site of DNA damage, which is important for efficient damage repair (modified from [52]). Click here for file

Additional file 7 **No evidence for Dnmt3a mediated *de novo *DNA methylation in the paternal pronucleus.** (A) Representative 2D projections of Z-stack images of control (B6xB6) and Dmnt3a maternally deleted oocytes fertilised by control sperm (Dnmt3a MAT KOxB6) late pronuclear stage embryos (PN3) simultaneously stained for DNA methylation (5mC- red) and hydroxymethylation (5hmC-green) showing no difference in both paternal loss of methylation and acquisition of hydroxymethylation, but a very obvious lack of maternal DNA methylation. Inset, merge with DNA staining (YOYO1) - blue. Scale bar 25 μm. f, female pronucleus; m, male pronucleus; pb, polar body. (B) Comparison of the changes of DNA methylation and hydroxymethylation between control (B6xB6), Dmnt3a maternally deleted (Dnmt3a MAT KOxB6) and AID null (AIDxB6) mid-late (PN3-PN5) zygotes. Box-and-whisker plots of the total paternal (male) pronucleus indirect immunofluorescence signal (3D imaging semi-quantification) for 5mC and 5hmC show no significant difference in either the levels of paternal DNA methylation (5mC) in Dnmt3a MAT KO fertilised oocytes relative to controls, on the left, or of hydroxymethylation (5hmC), on the right. ****(*P* <0.0001, ANOVA); ns (*P* >0.05, ANOVA). Click here for file
