# Supplementary figures and images for: Active demethylation in mouse zygotes involves cytosine deamination and base excision repair
Source: Epigenetics Chromatin. 2013 Nov 14;6:39. doi: 10.1186/1756-8935-6-39 (PMC4037648; doi:10.1186/1756-8935-6-39)

A

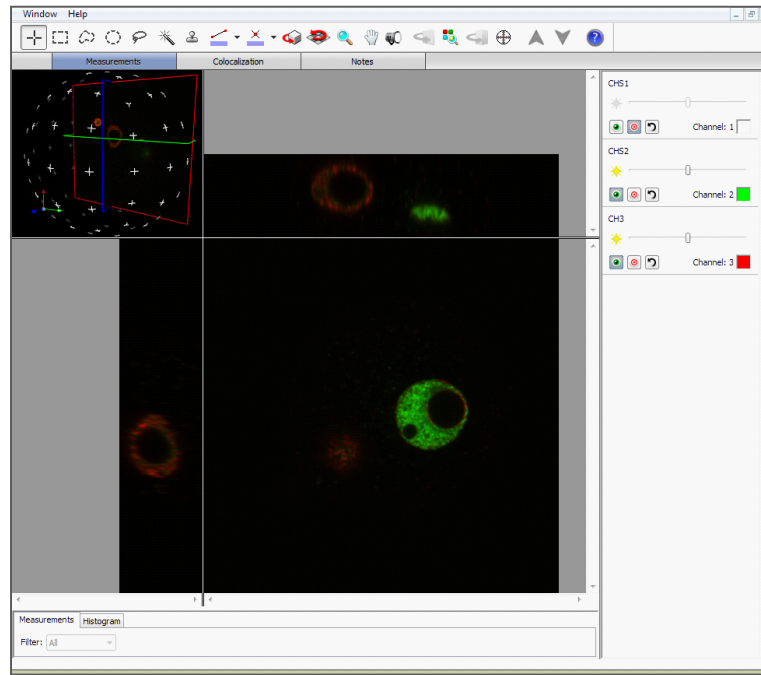

B

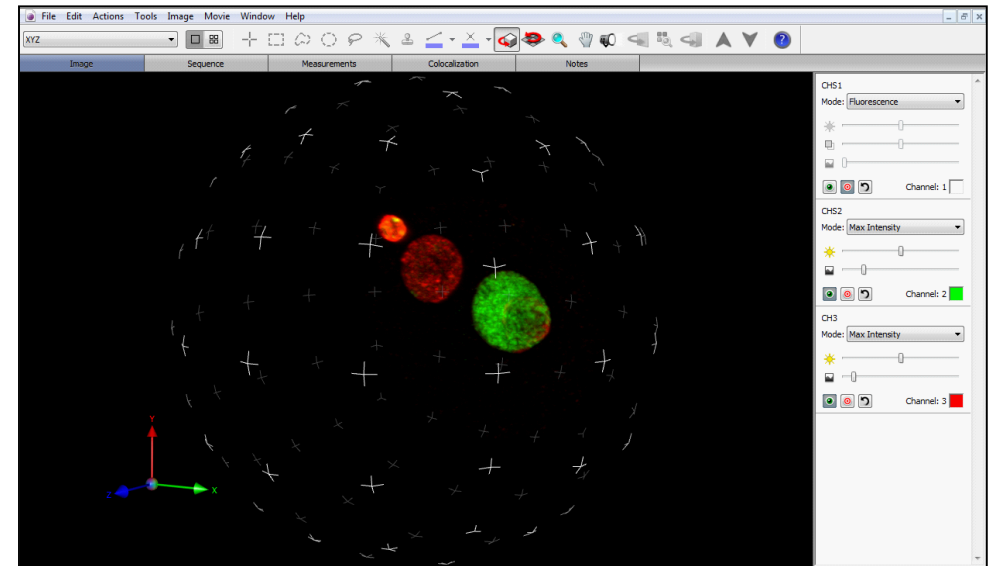

C

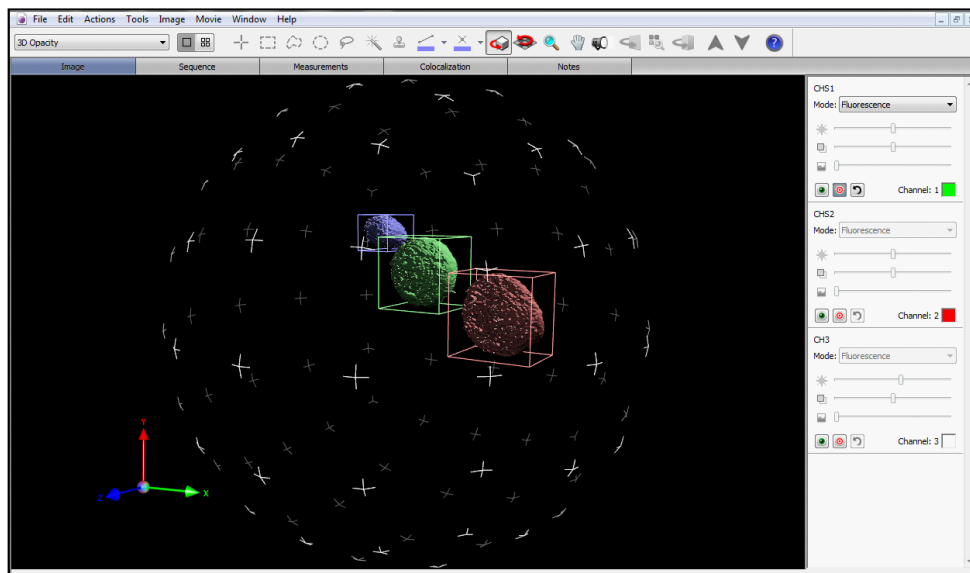

D

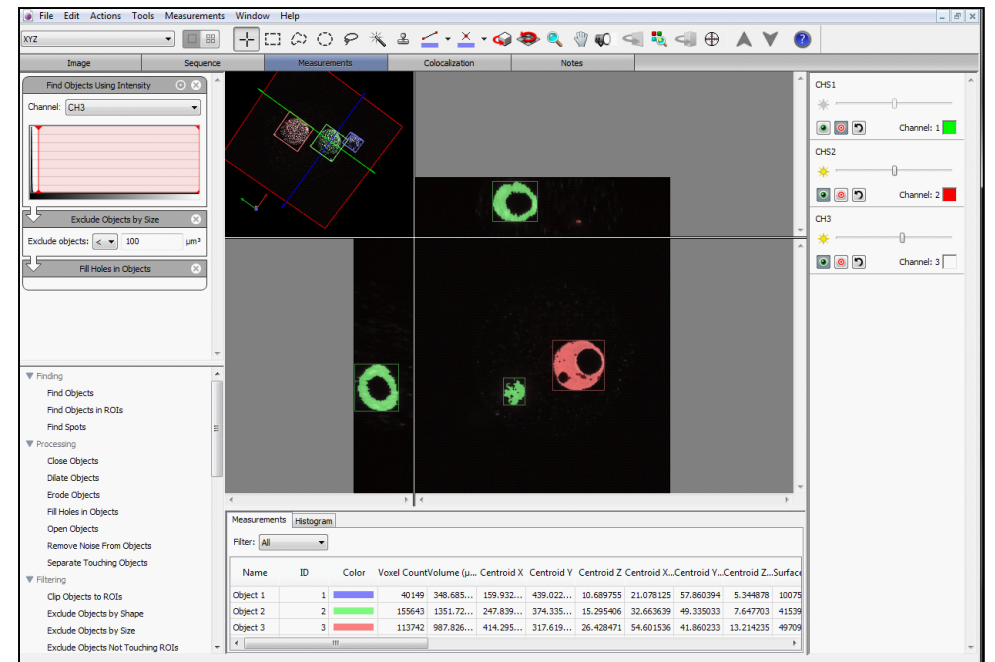

Supplement: Additional file 1 — 3D reconstruction of confocal image stacks and total fluorescence semi-quantification. Volocity 5.5 (Improvision) was used for 3D rendering and signal semi-quantification of each individual embryo Z-stack. (A) Screen-shot showing an XYZ view of a representative Z-stack. (B) Screen-shot showing the 3D rendering of the same Z-stack. (C) Screen-shot showing the regions of interest (ROIs) defined around each of the objects inside the sample, paternal pronucleus (red), maternal pronucleus (green) and polar body (blue). (D) Screen-shot showing the protocol to define the ROIs and subsequent computation of several measurements, including Sum signal intensity for each of the channels, used as a measure of the total signal for 5hmC (Channel: 2) and 5mC (Channel: 3). [file 1756-8935-6-39-S1.pdf]

A

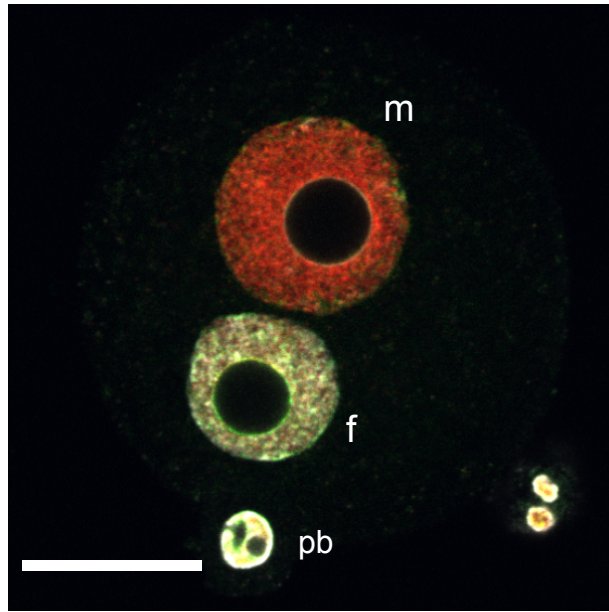

Control (DMSO)

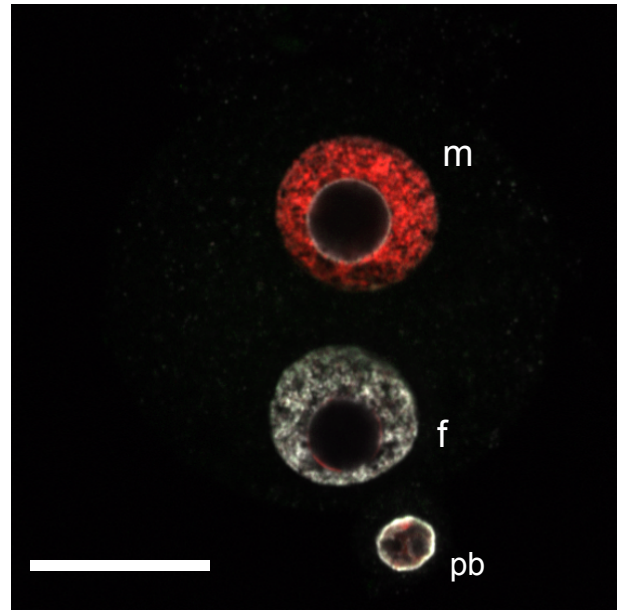

Aphidicolin

B

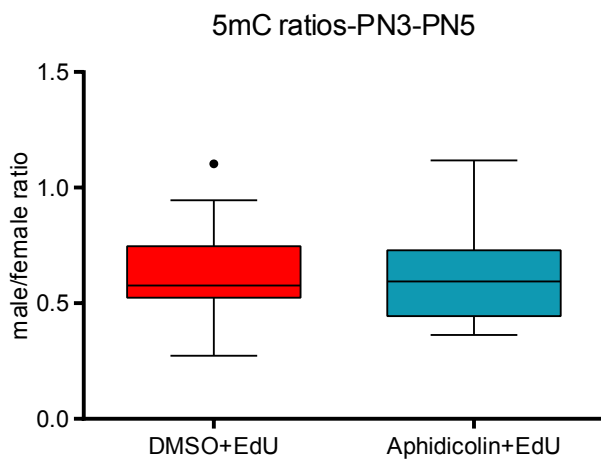

Unpaired t test  $P = 0.7842$

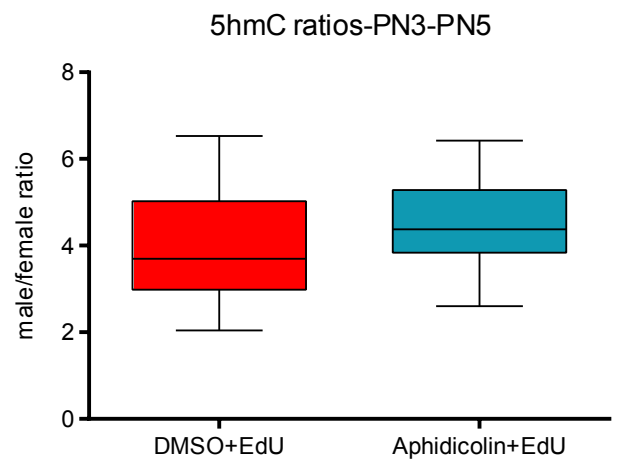

Unpaired t test  $P = 0.0748$

Supplement: Additional file 3 — Replication inhibition does not affect DNA methylation or hydroxymethylation paternal/maternal ratios in the zygote. Two independent replicates of at least 15 B6xB6 early fertilised oocytes were collected and incubated in M16 medium (M-7292-SIGMA) supplemented with either 2.5 μL/mL DMSO (control) or 2.5 μL/mL Aphidicolin (A4487-SIGMA) and cultured (37°C; 5%CO2) for 5 h. For replication analysis both groups (control-DMSO and Aphidicolin) were then transferred to a fresh same composition medium drop, to which 20 μM EdU (Click-iT™ EdU Alexa Fluor® 488, Invitrogen) was added, for a further 1 h (detection according to the manufacturer’s instructions). (A) Representative images of control (DMSO) and replication inhibited (Aphidicolin) embryos. Single optical slices. EdU-green; 5hmC-red; 5mC-white. Scale bar 25 μm. f, female pronucleus; m, male pronucleus; pb, polar body. (B) Comparison of the changes of DNA methylation and hydroxymethylation between control (DMSO) and replication inhibited (Aphidicolin) mid-late zygotes. Box-and-whisker plots of the total immunofluorescence signal (3D imaging semi-quantification) ratio between the paternal and maternal pronuclei (male/female ratio) for 5mC and 5hmC, respectively, showing no significant difference (unpaired t test) in the levels of methylation (left, P = 0.7842) or hydroxymethylation (right, P = 0.0748) in Aphidicolin-treated zygotes compared to controls (DMSO). [file 1756-8935-6-39-S3.pdf]

DAPI

$\alpha$ -Aid

merge

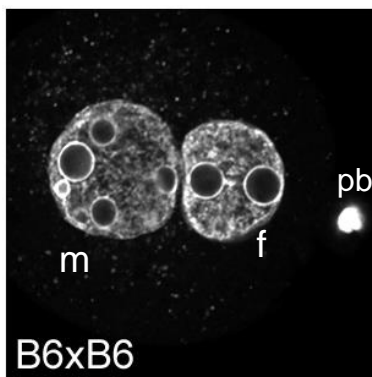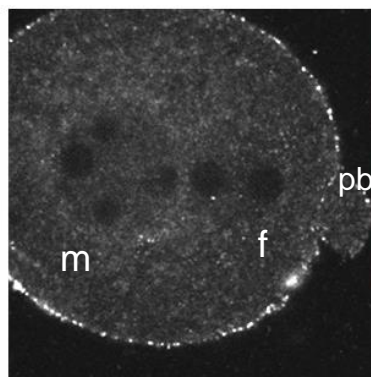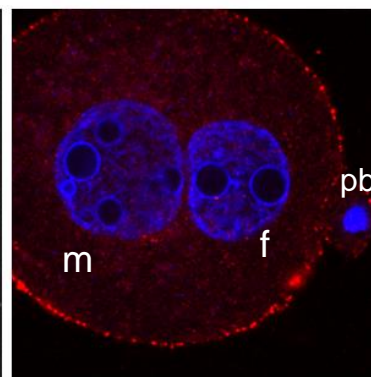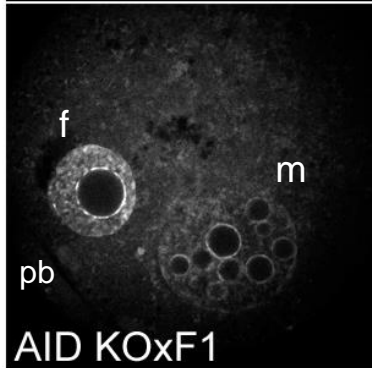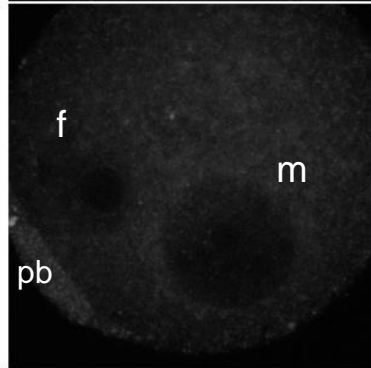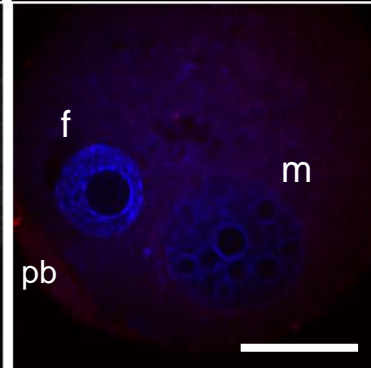

Supplement: Additional file 4 — AID is expressed in mouse oocytes and localises to the pronuclei. Wild-type controls (B6xB6) and AID null (AID KO x (C57Bl/6JxCBA)-F1) zygotes were stained with an antibody against AID (A-15, Santa Cruz Biotechnology) and DAPI. The control zygotes show a typical predominantly cytoplasmic localisation of AID protein, as has been described for B-cells (reviewed in [66]), but there is visible signal in both pronuclei that is completely absent in the AID null fertilised oocytes. Scale bar 25 μm. f, female pronucleus; m, male pronucleus; pb, polar body. [file 1756-8935-6-39-S4.pdf]

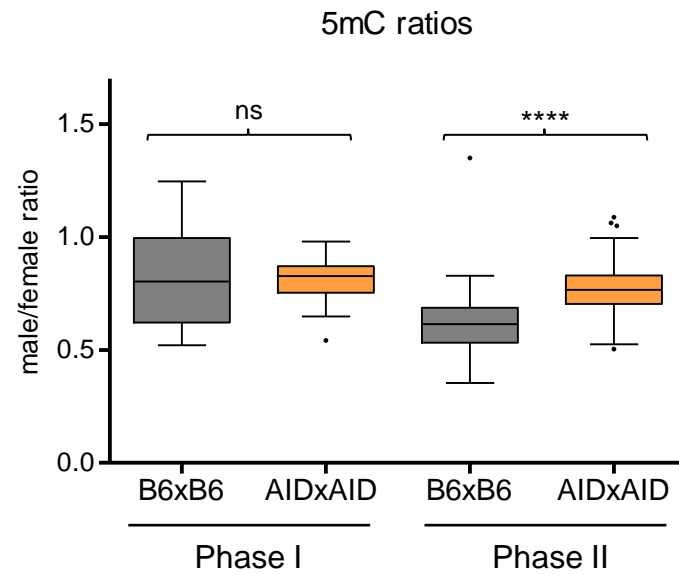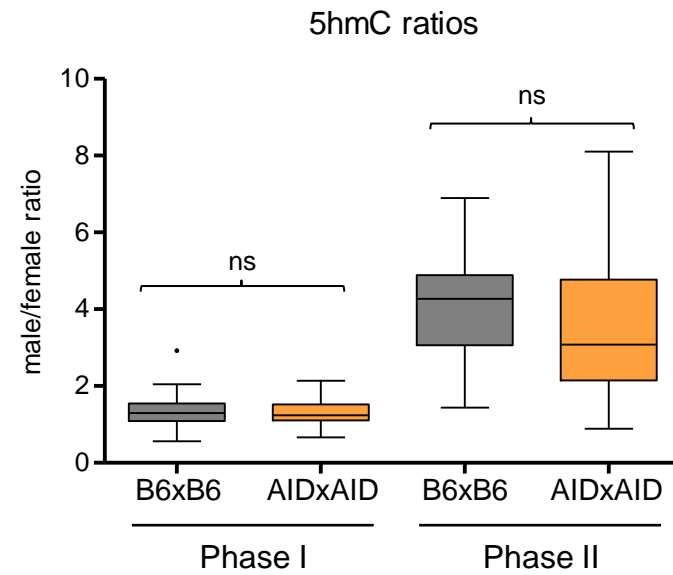

Supplement: Additional file 5 — AID null zygotes show no significant 5mC difference during Phase I of paternal demethylation. Comparison of the changes of DNA methylation and hydroxymethylation between wild-type control (B6xB6) and AID null (AIDxAID) Phase I (PN1-PN2) and Phase II (PN3-PN5) zygotes. Box-and-whisker plots of the total immunofluorescence signal (3D imaging semi-quantification) ratio between the paternal and maternal pronuclei (male/female ratio) for 5mC and 5hmC, respectively, showing, on the left, a very significant increase in the levels of paternal DNA methylation in Phase II but no significant difference in Phase I fertilised oocytes. No significant differences could be found in the levels of hydroxymethylation in AID null compared to wild-type in either Phase I or Phase II zygotes (on the right). ****(P <0.0001,Mann–Whitney test), ns (P >0.05, Unpaired t test). [file 1756-8935-6-39-S5.pdf]

A

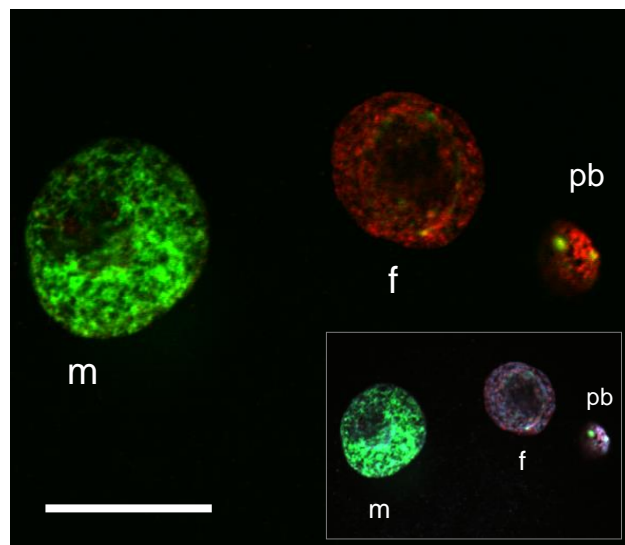

B6xB6

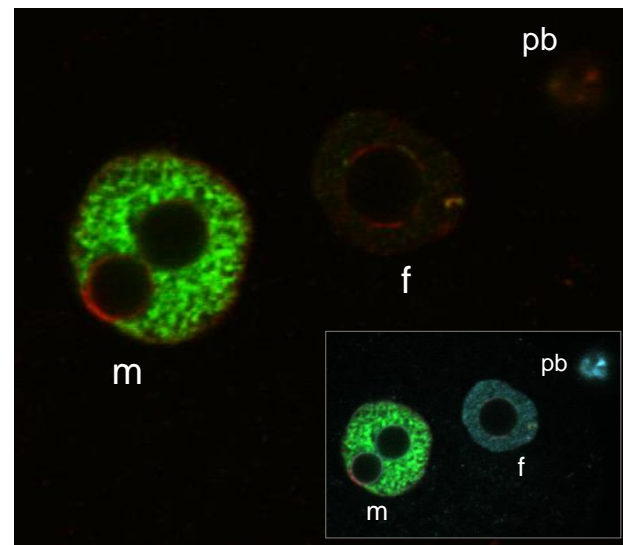

Dnmt3a MAT KOxB6

B

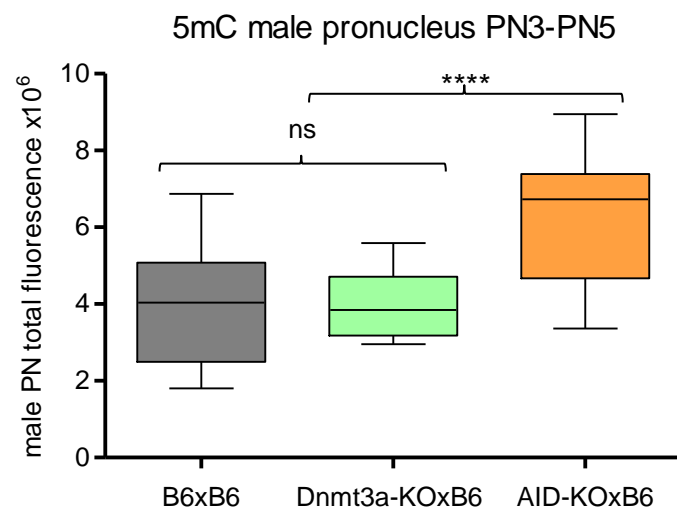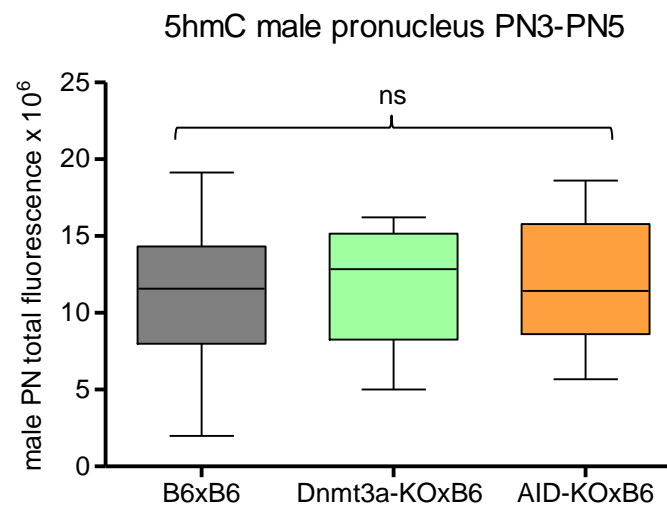

Supplement: Additional file 7 — No evidence for Dnmt3a mediated de novo DNA methylation in the paternal pronucleus. (A) Representative 2D projections of Z-stack images of control (B6xB6) and Dmnt3a maternally deleted oocytes fertilised by control sperm (Dnmt3a MAT KOxB6) late pronuclear stage embryos (PN3) simultaneously stained for DNA methylation (5mC- red) and hydroxymethylation (5hmC-green) showing no difference in both paternal loss of methylation and acquisition of hydroxymethylation, but a very obvious lack of maternal DNA methylation. Inset, merge with DNA staining (YOYO1) - blue. Scale bar 25 μm. f, female pronucleus; m, male pronucleus; pb, polar body. (B) Comparison of the changes of DNA methylation and hydroxymethylation between control (B6xB6), Dmnt3a maternally deleted (Dnmt3a MAT KOxB6) and AID null (AIDxB6) mid-late (PN3-PN5) zygotes. Box-and-whisker plots of the total paternal (male) pronucleus indirect immunofluorescence signal (3D imaging semi-quantification) for 5mC and 5hmC show no significant difference in either the levels of paternal DNA methylation (5mC) in Dnmt3a MAT KO fertilised oocytes relative to controls, on the left, or of hydroxymethylation (5hmC), on the right. ****(P <0.0001, ANOVA); ns (P >0.05, ANOVA). [file 1756-8935-6-39-S7.pdf]
