# Supplementary material for: Active demethylation in mouse zygotes involves cytosine deamination and base excision repair
Source: Epigenetics Chromatin. 2013 Nov 14;6:39. doi: 10.1186/1756-8935-6-39 (PMC4037648; doi:10.1186/1756-8935-6-39)

A

B6xB6

Group 1 (superovulation; 2010) – N= 41
Group 2 (natural mating; 2011) - N= 21
Group 3 (superovulation; 2012) - N= 24
Group 4 (superovulation and culture; 2013) - N= 13

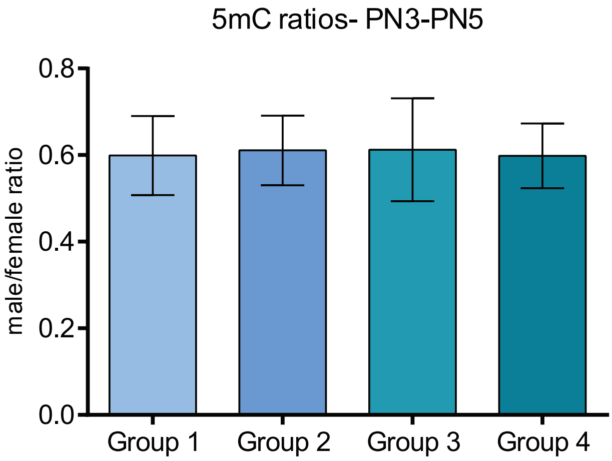

ANOVA P= 0.931

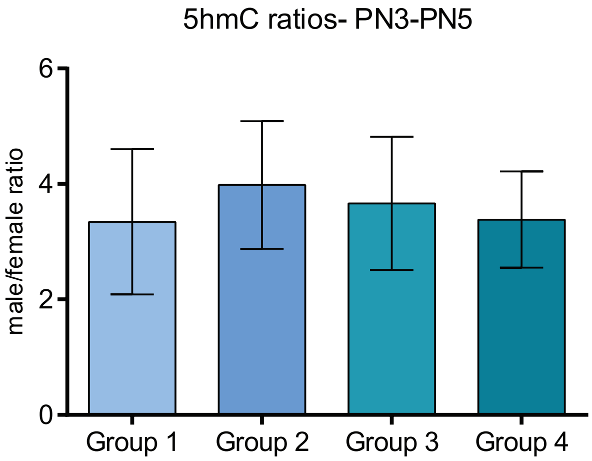

ANOVA P= 0.2017

B

E14- serum: 764 cells
E14- 2i: 760 cells

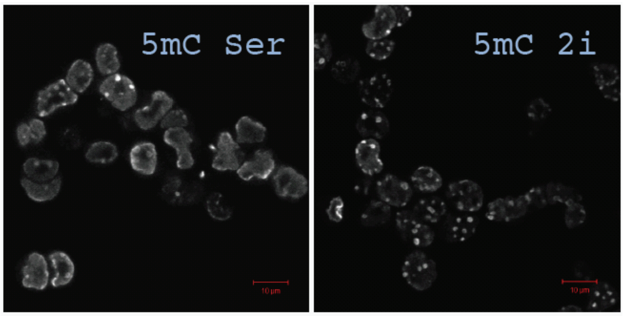

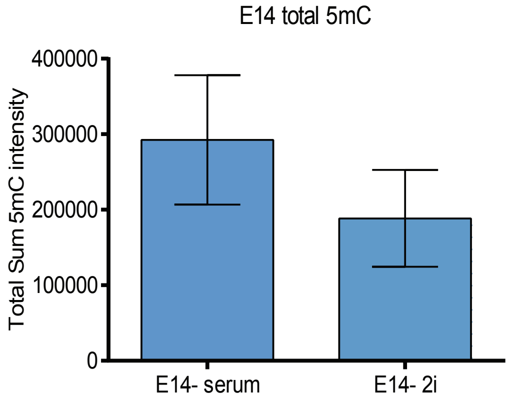

Mann-Whitney U: P< 0.0001

Mass spectrometry
measurement of global 5mC
Ficz et al. 2013

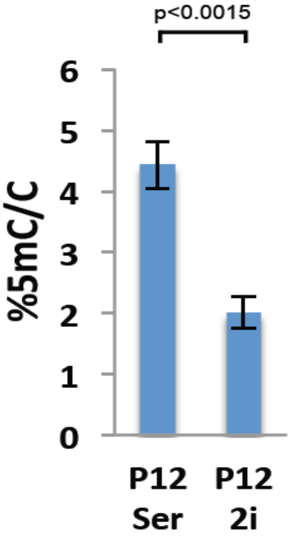

Supplement: Additional file 2 — Fluorescence semi-quantification protocol validation data. (A) Four independent samples of B6xB6 generated fertilised oocytes between PN3 and PN5 were evaluated using the optimized protocol for simultaneous staining of 5mC and 5hmC, 3D image acquisition and semi-quantification. Statistical analysis (ANOVA) shows no significant differences can be found between the four replicates. (B) Mouse embryonic stem cells (E14) were cultured in both serum and 2i conditions [57] and analysed for global DNA methylation levels by using the optimised immunofluorescence semi-quantification protocol (left) or by mass-spectrometry (right). Both methods are in agreement both qualitatively (E14 serum > E14 2i) and quantitatively (E14 serum 40% to 50% more methylated than E14 2i). [file 1756-8935-6-39-S2.pdf]
