# Supplementary material for: Active demethylation in mouse zygotes involves cytosine deamination and base excision repair
Source: Epigenetics Chromatin. 2013 Nov 14;6:39. doi: 10.1186/1756-8935-6-39 (PMC4037648; doi:10.1186/1756-8935-6-39)
Supplement: Additional file 6 — AID-mediated deamination and BER SP and LP pathways. Diagram of the SP and LP BER pathways for AID-mediated DNA deamination. AID has been shown in vitro and in E. coli to be capable of deaminating 5-methylcytosine (5mC), generating T-G mismatches and thus directly removing methylation from DNA (left hand-side), although the preferred substrate is cytosine, very efficiently generating U-G mismatches but having no direct effect on DNA methylation loss (right hand-side). After removal of the mismatched base (black circles) by a DNA glycosylase (TDG in the case of a T-G mismatch and UNG2 in the case of an U-G mismatch) and incision by apurinic/apyrimidinic endonuclease (APE1), BER may proceed by the SP repair or by the LP repair. SP BER replaces a single nucleotide by polymerase β and the newly synthesized DNA sealed by DNA ligase III/X-ray cross-complementing group 1 (XRCC1) heterodimer. LP BER inserts two to 13 nucleotides by concordant action of polymerase δ, proliferating cell nuclear antigen (PCNA), flap endonuclease 1 and DNA ligase I. In this case, any methylated cytosines adjacent to the generated U-G mismatch would be replaced by new cytosines and, if not subsequently de novo methylated, the original methylated state would be lost resulting in demethylation. Poly ADP Ribose Polymerase 1 (PARP1), which binds to and is activated by DNA strand breaks, has been implicated in LP repair promoting the rapid recruitment of PAR-binding proteins to the site of DNA damage, which is important for efficient damage repair (modified from [52]). [file 1756-8935-6-39-S6.pdf]

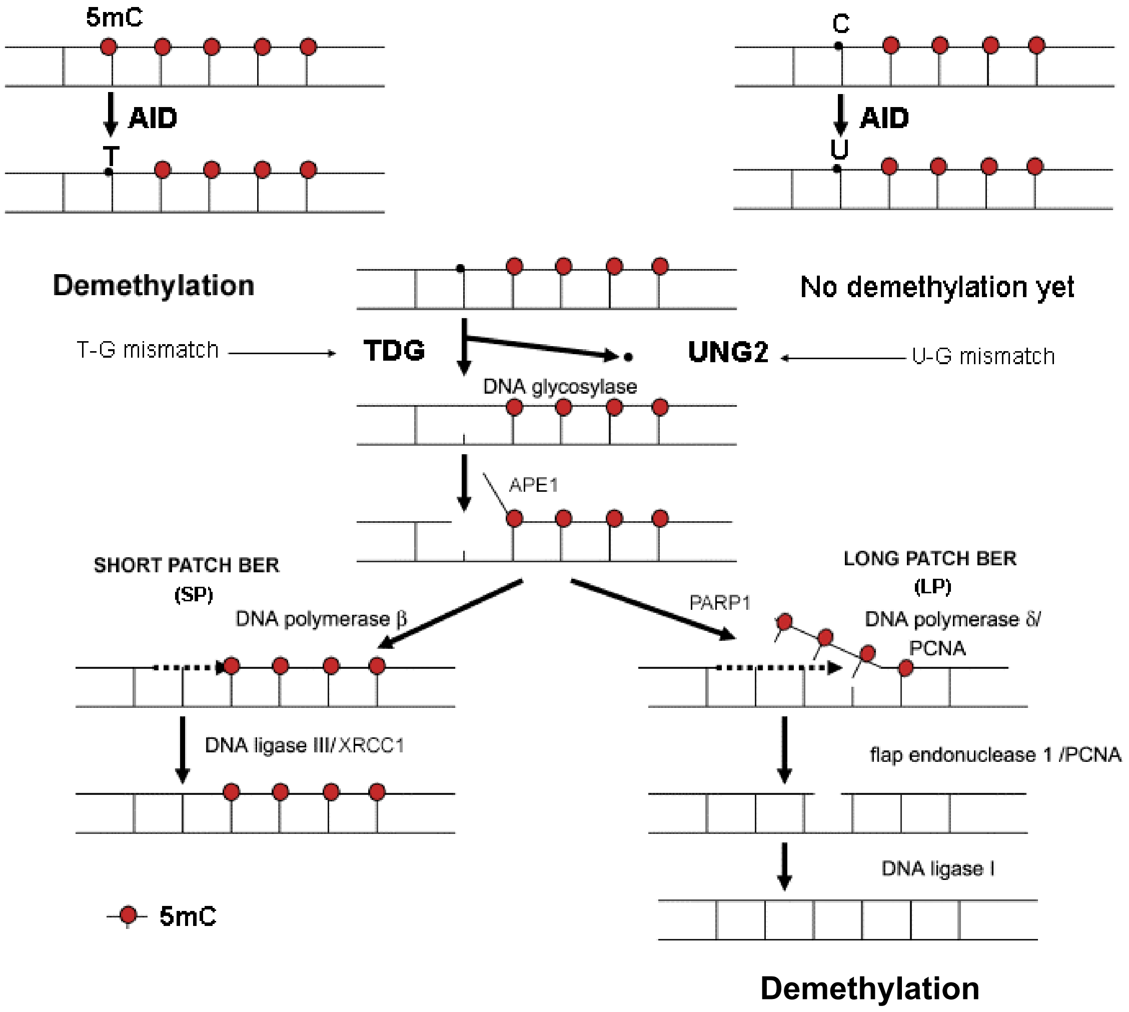

5mC
AID
T
C
AID
U
Demethylation
No demethylation yet
T-G mismatch
TDG
UNG2
U-G mismatch
DNA glycosylase
APE1
SHORT PATCH BER
(SP)
LONG PATCH BER
(LP)
PARP1
DNA polymerase β
DNA polymerase δ/
PCNA
DNA ligase III/ XRCC1
flap endonuclease 1 /PCNA
DNA ligase I
5mC
Demethylation
